# Global transcription machinery engineering in *Yarrowia lipolytica*

**DOI:** 10.1093/femsyr/foaf023

**Published:** 2025-05-08

**Authors:** Ewelina Celińska, Yongjin J Zhou

**Affiliations:** Department of Biotechnology and Food Microbiology, Poznan University of Life Sciences, ul. Wojska Polskiego 48, 60-637 Poznań, Poland; Division of Biotechnology, Dalian Institute of Chemical Physics, Chinese Academy of Sciences, Dalian 116023, China; CAS Key Laboratory of Separation Science for Analytical Chemistry, Dalian Institute of Chemical Physics, Chinese Academy of Sciences, Dalian 116023, China; Dalian Key Laboratory of Energy Biotechnology, Dalian Institute of Chemical Physics, Chinese Academy of Sciences, Dalian 116023, China; State Key Laboratory for Quality Ensurance and Sustainable Use of Dao-di Herbs, Beijing 100700, China

**Keywords:** global metabolic engineering, transcription factors, yeast, complex traits

## Abstract

Global transcription machinery engineering (gTME) is a strategy for optimizing complex phenotypes in microbes by manipulating transcription factors (TFs) and their downstream transcriptional regulatory networks (TRN). In principle, gTME leads to a focused but comprehensive optimization of a microbe, also enabling the engineering of nonpathway functionalities, like stress resistance, protein expression, or growth rate. A link between a TF and a desired phenotype is to be established for a rationally designed gTME. For use in a high-throughput format with extensive libraries of TRN-engineered clones tested under multiple conditions, well-developed culturing and analytical protocols are needed, to reveal the pleiotropic effects of the TFs. This mini-review summarizes the gTME strategies and TFs described under different contexts in *Yarrowia lipolytica*. The outcomes of the gTME strategy application are also addressed, demonstrating its effectiveness in engineering complex, industrially relevant traits in *Y. lipolytica*.

## Teaser

Global transcription machinery engineering using TFs allows complex traits engineering in yeast.

## Principles behind the global transcription machinery engineering

**Figure 1.**
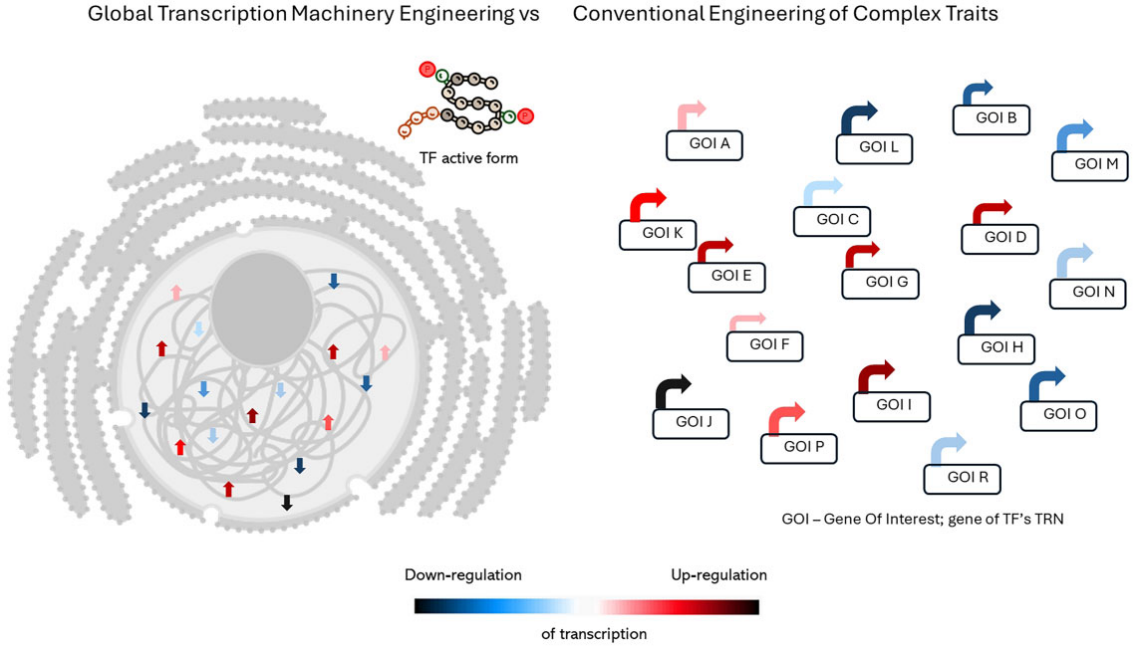
A simplified global transcription machinery engineering concept compared to a conventional strategy for complex traits engineering. The presented idea is greatly simplified, presuming the TF’s TRN does not overlap with another TF. Color code—the level of up-/down-regulation according to a legend.

The basic functioning of a biological entity does not rely on an “over-production phenotype.” Yet, the latter is required for a truly industrially relevant biotechnological process. A phenotype is created by the interplay between environmental factors and genetics. For an adequate, industrially relevant, genetic background, multiple (dozens–hundreds) genes must be harmonized. Technically, this goal can be achieved by targeting molecular identities operating at higher event levels, like kinases/phosphatases acting within signaling cascades or transcription factors (TFs) governing transcriptional regulatory networks (TRNs). In that case, only a limited number of genetic manipulations is required to reach a global-level fine-tuning of a microbe toward a high-producing phenotype (Fig. 1). A TRN is defined as a regulatory interaction between a given TF (trans-element) and its target gene (cis-element) directly involved in a specific biological process (He and Tan 2016, Su et al. 2022). Skillful manipulation of TRNs leads to a focused but comprehensive optimization of a microbe, eliciting the most adequate response to a researcher-defined target. Phenotype engineering by the manipulation of TRNs may also include nonpathway-based functionalities, such as stress tolerance or growth rate, which are highly relevant in industrial contexts (Alper and Stephanopoulos 2007, Lanza and Alper 2011).

**Figure 2.**
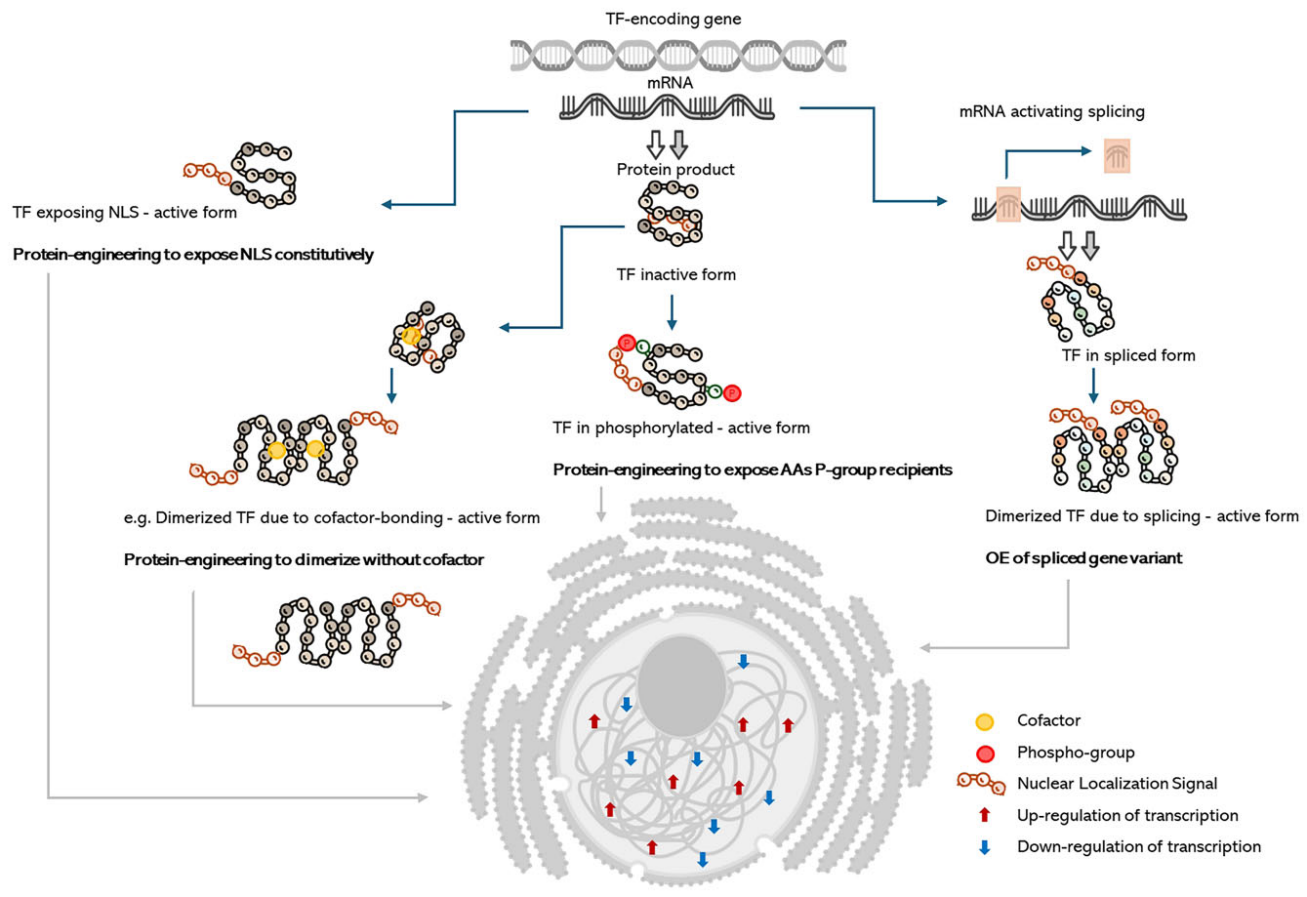
Selected, exemplary mechanisms of TF’s activation and proposed strategies from the protein engineering area for synthetic constitutive activation of the TF (bolded). AA—amino acid; P—phosphate group, and NLS—nuclear localization signal.

Notably, TFs respond to various stimuli by altering expression patterns and activation status (Peñalva and Arst 2002, Cornet and Gaillardin 2014). Hence, it is insufficient to just manipulate TF-encoding genes to modulate their normal transcriptional regulation by overexpression (OE) or knock-out (KO). It is necessary to consider the operative mechanism of the TFs, by implementing accurate steps like (i) continuous targeting of the TF to the nucleus, (ii) expression of the TF in its spliced/alternatively spliced form, (iii) modifying the TF’s polypeptide sequence to expose/bury the key residues that receive the phosphate group, (iv) challenge the TF-overexpressing strain with an environmental perturbation/exposure to a cofactor that activates the TF, and so on (Fig. 2).

## Global transcriptional machinery Engineering in yeast

The concept of using TRNs for engineering complex traits in yeast was first proposed by Alper et al. (2006). Later, Alper and Stephanopoulos (2007) named this approach “global Transcription Machinery Engineering” for optimizing complex target phenotypes in microbes. In their pioneering study (Alper et al. 2006), the authors mutated the TF Spt15 and selected mutants with enhanced tolerance to ethanol and conversion of glucose to ethanol. Technically, several amino acid (AA) substitutions were introduced in Spt15, conferring the desired phenotype to *Saccharomyces cerevisiae*. TF Spt15 was mutated to develop an *S. cerevisiae* strain exhibiting reduced ethanol yield by enhancing carbon flux toward CO_2_, biomass, and glycerol production (Du et al. 2020). In total, five AA substitutions in Spt15 contributed to a vast remodeling of metabolism and the development of target functionalities. In a varied display of global transcription machinery engineering (gTME) in yeast, deletion of the TF *CAT8* triggered an increased conversion of xylose to ethanol in *Ogataea polymorpha* by modulating the expression of genes involved in central carbon catabolism (Ruchala et al. 2017).

Huang et al. (2017) detailed how massive molecular remodeling of a yeast cell is caused by the synthesis of a recombinant protein (rProt). *Inter alia*, they noted that the process is innately associated with oxidative stress, mainly originating from the endoplasmic reticulum (ER)-located oxidative folding. They proposed that oxidative stress attenuation can be achieved by either the activation of Unfolded Protein Response (UPR) and/or the enhancement of protein folding capacity. The most common strategy for the activation of UPR is *via* the OE of the TF *HAC1*^1^. Hac1^1^ is involved in the direct regulation of ER-resident events and restoring ER homeostasis. The OE of *HAC1* specifically improved the secretion of rProt in *S. cerevisiae* (Valkonen et al. 2003, Duan et al. 2019), *Pichia pastoris* (now *Komagataella phaffi*) (Gasser et al. 2007b, Guerfal et al. 2010, Liu et al. 2020), and in *Yarrowia lipolytica* (Korpys-Woźniak et al. 2021, Korpys-Woźniak and Celińska 2023). Other studies demonstrated the usefulness of the Hsf1 TF in this regard. Hsf1 is a key regulator of the heat shock response, governing the protein folding and chaperoning capacity of a cell (Lindquist and Craig 1988, Sorger 1991). The regulome (a total of the deregulated genes) of the Hsf1 in *S. cerevisiae* was mainly represented by molecular chaperones involved in protein folding, thus preventing the accumulation of misfolded or aggregated polypeptides (Hou et al. 2013). The OE of a mutant *HSF1*-R206S (constitutively active form) triggered the elevated production of native and recombinant proteins in *S. cerevisiae* (Hou et al. 2013).

The detailed characteristics of *S. cerevisiae* cells at the system level (Huang et al. 2017) evidenced the responsiveness of the TF Hap1 to enhanced rProt synthesis. Hap1 is implicated in regulating aerobic metabolism in *S. cerevisiae*. It is involved in sensing O_2_ levels *via* the heme signaling pathway, and in response activates the oxidative stress response-associated genes (Zhang and Hach 1999). The OE of *HAP1* in *S. cerevisiae* attenuated the negative impacts of oxidative stress and elevated the production of the target rProt (Martínez et al. 2016). Recently, the synthetic activation of a general stress response TF, Msn4, and its synthetic version synMsn4, alone or in combination with constitutively active Hac1 or ER chaperones triggered an over 4-fold enhancement in antibody production (Zahrl et al. 2023). Notably, a combination of the two global and constitutively active regulators, Hac1 and Msn4, produced the best results.

The literature provides many successful examples of gTME for complex traits engineering in conventional and nonconventional yeast species, demonstrating the high potential and efficiency of this approach. Of note, TRNs in different yeast species may display distinct characteristics, as yeast metabolism is adapted to other functionalities. For example, a lipid-accumulation-related TRN is expected to be more extensive and robust in a nonconventional yeast species, *Y. lipolytica*, than in a sugar-utilizer and ethanol-producer *S. cerevisiae*. In fact, the TRN of nonconventional yeast in many cases was better applicable to industrially relevant traits, making them attractive hosts/donors.

## TFs in *Y. lipolytica*

In *Y. lipolytica*, a yeast species of interest, knowledge regarding TFs largely addressed the involvement of different genes for specific qualities, like filamentation, lipid accumulation, stress resistance, and so on. They revealed the transcriptional-deregulation pattern of a TF-encoding gene under specific conditions or modulation of a trait of interest in the absence (due to KO) or OE of a gene. In the following sections, we review data on the TFs studied in *Y. lipolytica* collected, to date. They are also summarized in Table 1. Of note, due to the influence of many TFs on a broad spectrum of cellular responses, it is impossible to assign a single specific role. Therefore, the following assignment of individual TFs to specific biological processes may result only due to the context in which the TF was studied and found to be associated.

**Table 1.** Selected ylTFs, discussed in this mini-review. Based on Gorczyca et al. (2024), modified.

Yali number	Assigned name	Putative/known function	Regulome/iModulon available?	YaliFunTome (a direct link to OE phenotype across 72 conditions)
*B12716*	*HAC1*	Transcriptional activator of genes involved in ER-based UPR	Regulome (Korpys-Woźniak and Celińska 2023)	https://sparrow.up.poznan.pl/tsdatabase/?page=gene&name=TF006#growth
*E31757*	*BRG1*	Biofilm regulator 1, induces lipid synthesis/accumulation		https://sparrow.up.poznan.pl/tsdatabase/?page=gene&name=TF053#growth
*D14520*	*SKN7*	TF involved in the activation of osmotic and oxidative stress		https://sparrow.up.poznan.pl/tsdatabase/?page=gene&name=TF033#growth
*D07744*	*YAP-like*	TF involved in pH-dependent dimorphic transition and maintaining redox balance		https://sparrow.up.poznan.pl/tsdatabase/?page=gene&name=TF029#growth
*D20482*	*GZF1*	Inducer of the nitrogen catabolite repression (NCR) genes		https://sparrow.up.poznan.pl/tsdatabase/?page=gene&name=TF037#growth
*E13948*	*HSF1*	Heat shock TF		https://sparrow.up.poznan.pl/tsdatabase/?page=gene&name=TF068#growth
*D05041*	*KLF1*	Krueppel-like factor 15, regulates the expression of genes for gluconeogenic and AA-degrading enzymes		https://sparrow.up.poznan.pl/tsdatabase/?page=gene&name=TF126#growth
*A16841*	*AZF1*	Asparagine-rich zinc finger protein, regulates carbon metabolism in yeast and cell wall organization		https://sparrow.up.poznan.pl/tsdatabase/?page=gene&name=TF124#growth
*F05896*	*DEP1*	Part of the Rpd3C(L) histone deacetylase complex (HDAC). Transcriptional modulator involved in regulation of structural phospholipid biosynthesis genes		https://sparrow.up.poznan.pl/tsdatabase/?page=gene&name=TF128#growth
*C19151*	*CAT8*	CATabolite repression TF 8. Inducer of gluconeogesis, maintains energy homeostasis, presumed to regulate formate dehydrogenases expression	iModulon (Kerssemakers et al. 2023a, b)	https://sparrow.up.poznan.pl/tsdatabase/?page=gene&name=TF122#growth
*F21923*	*ADR1*	Alcohol dehydrogenase II synthesis regulator, inducer of genes involved in alternative carbon utilization upon glucose starvation. Repressor of ERY-induced expression of EYK1 in the presence of glucose or glycerol	iModulon (Kerssemakers et al. 2023a, b)	https://sparrow.up.poznan.pl/tsdatabase/?page=gene&name=TF116#growth
*D20460*	*LAC9*	LACtose regulatory protein, controls induction of the lactose-galactose regulation		https://sparrow.up.poznan.pl/tsdatabase/?page=gene&name=TF036#growth
*B14443*	*JMC2*	JmjC domain family histone demethylase, promotes global demethylation of H3K4		https://sparrow.up.poznan.pl/tsdatabase/?page=gene&name=TF009#growth
*D02783*	*DAL81*	Positive regulator of genes in multiple nitrogen degradation pathways, involved in nitrogen catabolite activation of transcription from RNA polymerase II promoter		https://sparrow.up.poznan.pl/tsdatabase/?page=gene&name=TF118#growth
*B19602*	*MGF2*	Mycelial growth factor 2. TF of known roles in the dimorphic transition		https://sparrow.up.poznan.pl/tsdatabase/?page=gene&name=TF010#growth
*B21582*	*MHY1/MSN2*	Msn2/Msn4-like protein, a key regulator of yeast-to-hypha dimorphic transition but not stress resistance, regulates both alkaline-pH and glucose-induced filamentation. regulated by the kinase Rim15p, which itself is repressed by the Tor nitrogen signaling pathway	Regulome (Pomraning et al. 2018, Wang et al. 2018, Wu et al. 2020, Shu et al. 2021)	https://sparrow.up.poznan.pl/tsdatabase/?page=gene&name=TF095#growth
*D01573*	*MGF1*	Mycelial growth factor. Potential driver of the transition between morphological phases		https://sparrow.up.poznan.pl/tsdatabase/?page=gene&name=TF115#growth
*B05038*	*ZNC1*	Zinc finger transcriptional factor, regulates the yeast-to-hypha transition in the dimorphic yeast		https://sparrow.up.poznan.pl/tsdatabase/?page=gene&name=TF131#growth
*F17886*	*GZF2*	GATA–binding zinc finger TF 2, inducer of NCR, essential for growth on simple nitrogen sources	Regulome (Pomraning et al. 2017)	NA
*C22682*	*GZF3*	GATA-zinc finger TF 3, repressor of NCR	Regulome (Pomraning et al. 2017)	https://sparrow.up.poznan.pl/tsdatabase/?page=gene&name=TF072#growth
*E05555*	*GZF4*	GATA-zinc finger TF 4. Putative: inducer of NCR		https://sparrow.up.poznan.pl/tsdatabase/?page=gene&name=TF041#growth
*E16577*	*GZF5*	Nongenuine GATA-zinc finger TF		https://sparrow.up.poznan.pl/tsdatabase/?page=gene&name=TF109#growth
*E03410*	*ERT1-2*	Positive regulator of gluconeogenesis		https://sparrow.up.poznan.pl/tsdatabase/?page=gene&name=TF040#growth
*E27742*	*GCN4*	General control nonderepressible 4 TF. Key transcriptional activator of AA biosynthesis genes		https://sparrow.up.poznan.pl/tsdatabase/?page=gene&name=TF091#growth
*F17424*	*HAP1*	TF responsible for oxygen sensing and signaling		https://sparrow.up.poznan.pl/tsdatabase/?page=gene&name=TF120#growth
*F01562*	*EUF1*	TF mediating expression of erythritol synthesis genes	Regulome (Rzechonek et al. 2024)	https://sparrow.up.poznan.pl/tsdatabase/?page=gene&name=TF054#growth
*C16863*	*SKO1*	*ATF/CREB* family TF, repressor that mediates *HOG* pathway-dependent regulation of osmotic stress response, involved in protection from oxidative damage		https://sparrow.up.poznan.pl/tsdatabase/?page=gene&name=TF022#growth
*C13750*	*MSN4*	General stress response, regulates tolerance to acid-induced stress. Regulates genes involved in the antioxidant cellular response		https://sparrow.up.poznan.pl/tsdatabase/?page=gene&name=TF107#growth
*B13640*	*RIM101*	pH-response TF, regulator of alkaline-induced filamentation	Regulome (Shu et al. 2021)	NA
*C12364*	*NRG1*	*NRG1* repressor of erythritol utilization genes, plays a minor role in repression of filamentation		https://sparrow.up.poznan.pl/tsdatabase/?page=gene&name=TF018#growth
*C18645*	*ARO80*	Transcription activator required for the expression of genes involved in the catabolism of aromatic AAs		https://sparrow.up.poznan.pl/tsdatabase/?page=gene&name=TF121#growth
*B08206*	*CRF1*	Copper resistance protein transcriptional regulator		https://sparrow.up.poznan.pl/tsdatabase/?page=gene&name=TF077#growth
*E18304*	*ERT1-1*	Transcription activator of gluconeogenesis		https://sparrow.up.poznan.pl/tsdatabase/?page=gene&name=TF127#growth
*E07942*	*MIG1*	Controls genes involved in beta-oxidation, involved in carbon catabolite repression	Regulome (Wang et al. 2013)	https://sparrow.up.poznan.pl/tsdatabase/?page=gene&name=TF042#growth
*A18469*	*HOY1*	Homeobox protein, a positive regulator of hyphae formation		https://sparrow.up.poznan.pl/tsdatabase/?page=gene&name=TF099#growth
*F05104*	*TFIIIA*	*PZF1* general TF IIIA		https://sparrow.up.poznan.pl/tsdatabase/?page=gene&name=TF057#growth
*D12628*	*POR1*	Primary oleate regulator 1–transcriptional activator regulating genes involved in fatty acid utilization		https://sparrow.up.poznan.pl/tsdatabase/?page=gene&name=TF079#growth
*C02387*	*YAS1*	TF essential for cytochrome p450 induction in response to alcanes, heteromeric Yas1p/Yas2p complex TF		https://sparrow.up.poznan.pl/tsdatabase/?page=gene&name=TF083#growth
*B08734*	*REI1*	Cytoplasmic pre-60S factor *REI1* involved in maturation of the ribosomal 60S subunit		https://sparrow.up.poznan.pl/tsdatabase/?page=gene&name=TF084#growth
*A19778*	*MBP1*	MluI-box binding protein, involved in the regulation of cell cycle progression from G1 to S phase	Regulome (Pomraning et al. 2018) iModulon (Kerssemakers et al. 2023a, b)	https://sparrow.up.poznan.pl/tsdatabase/?page=gene&name=TF090#growth
*F25861*	*RPN4*	*RPN4* TF regulating proteasomal genes		https://sparrow.up.poznan.pl/tsdatabase/?page=gene&name=TF108#growth
*C12639*	*SWI6*	*SWI6* part of a complex involved in cell-cycle-dependent transcription. *SWI4* and *SWI6* are required for formation of the cell-cycle box factor-DNA complex	iModulon (Kerssemakers et al. 2023a, b)	https://sparrow.up.poznan.pl/tsdatabase/?page=gene&name=TF111#growth

### Filamentation and associated TFs

Due to its dimorphic nature, *Y. lipolytica* has been extensively studied in the context of morphological shift between filamentous and ovoid morphotypes (Barth and Gaillardin 1997, Ruiz-Herrera and Sentandreu 2002, Szabo and Štofaníková 2002, Kawasse et al. 2003, Bellou et al. 2014, Braga et al. 2016, Timoumi et al. 2017, Bankar et al. 2018). Filamentation is generally acknowledged as a marker of cellular stress, inflicted by external or internal factors; recently reviewed by Celińska (2022). Thus, TFs involved in filamentation are also frequently implicated in stress responses with different origins.

Many TFs have been implicated in dimorphic shift, including Mhy1/Msn2 (*B21582* ^2^) (Hurtado and Rachubinski 1999, Pomraning et al. 2018, Wu et al. 2020, Shu et al. 2021), Yap-like *D07744* (Morales-Vargas et al. 2012), Rim101/*B13640* under alkaline pH (Shu et al. 2021). Fts2/*E23287* (Chen et al. 2022), Znc1/*B05038* (Martinez-Vazquez et al. 2013), Bmh1/*F18590* (Hurtado and Rachubinski 2002), Hoy1/*A18469* (Torres-Guzmán and Domínguez 1997), Tec1/*F05632* (Zhao et al. 2013), the mycelial growth factors Mgf1/*D01573* and Mgf2/*B19602*, Mig1/*E07942* under glucose starvation (Wang et al. 2013), as well as for a Swi6/*C12639*–Mbp1/*A19778* complex (Pomraning et al. 2018). Most studies applied canonical phenotypic analysis of the KO/OE mutants, proving the direct involvement of a specific TF in dimorphic transition. The other studies applied transcriptomics to identify the differently expressed genes.

Some TFs involved in invasive growth were also studied for their role in stress response, frequently demonstrating the link between these qualities. For example, a primary study on Mhy1 (Msn2) in *Y. lipolytica* demonstrated its upregulation during dimorphic shift, neutral-alkaline pH, and the presence of glucose (Hurtado and Rachubinski 1999). Based on this observation, and similarity to *S. cerevisiae*’s zinc finger-type Msn2/4 TF, Mhy1 was identified as a key positive regulator of general stress responses and alkaline- or glucose-induced filamentation (Shu et al. 2021). In contrast, testing of *Y. lipolytica* strain under 72 varied conditions (Gorczyca et al. 2024), indicated that even under the infliction of relevant stress factors, OE of *MHY1* did not markedly promote the strain’s growth—resistance to these stress conditions (please refer to the YaliFunTome database: https://sparrow.up.poznan.pl/tsdatabase/). Consistently, Wu et al. (2020) evidenced that, in this species, Mhy1 is not required for increased stress resistance but plays a role only in dimorphic transition. Likewise, the nonimplication of Mhy1in stress response was confirmed by Konzock and Norbeck (2020). Hence, developing *Δmhy1* is a common strategy for abolishing problematic filamentation without impairing the resistance of *Y. lipolytica* to stress factors (Vidal et al. 2023).

TFs with a characterized regulome are of special interest for devising gTME strategies. As a logical consequence of extensive research into *Y. lipolytica*’s dimorphic transition, the TFs primarily regulating this phenomenon were studied extensively, including their regulomes. An elegant display of such insightful research was provided by Pomraning et al. (2018) while studying the genetic background of a “smooth” (not filamenting) phenotype in *Y. lipolytica*. The authors ran comparative genomics and transcriptomics, and the following functional studies focused on *MHY1*. High-throughput genomics enabled the reliable identification of multiple genes involved in the dimorphic shift. Transcriptomics enabled the identification of genes deregulated in the “smooth” strain. Promoter sequence analysis revealed the following DNA motifs shared by the genes: ACGCG for upregulation, and CCCCT for downregulation. Consequently, compatible TFs including Mhy1/Msn2, specific for an “ACGCG motif,” and the cell-cycle regulatory proteins Mbp1 and Swi6, specific for a “CCCCT” motif were deduced. Functional studies proved that Mhy1/Msn2 expression promotes hyphal growth, while Swi6/Mbp1 forms a complex that regulates the G1/S phase transition and retention in an ovoid morphotype. Importantly, Mbp1 is one of several TFs with delimited iModulons (Kerssemakers et al. 2023a). An iModulon is a set of independently coregulated genes. They are unraveled and structured from high-quality RNA-seq datasets using a machine-learning approach. The Mbp1’s iModulon was found to be responsive to CO_2_ levels and promoted the activation of cell cycle progression.

Mhy1’s regulome was also extensively characterized in the context of lipid accumulation (Wang et al. 2018) (showing its relevance to Section 3.2). Indeed, the Mhy1 regulome covered 24.7% (1597/6472) of *Y. lipolytica*’s annotated genes, including lipid, AA, and nitrogen metabolism, as well as cell cycle progression. In the *Δmhy1* strain, the enhanced carbon flux toward lipid biosynthesis was accompanied by a reduced flux through AA biosynthesis. Consistent with the results by Pomraning et al. (2018), an interplay between cell cycle progression and filamentation was observed, which was enhanced in *Δmhy1*. Wang et al. (2018) comprehensively illustrated the operation of Mhy1’s regulome and deduced the expected outcomes of gTME exploiting *MHY1*. Wu et al. (2020) provided further detailed characterization of Mhy1, the gene and promoter structure, and the regulome. Here, the transcriptome profiles of the *Δmhy1* mutant and *MHY1*-OE strains were compared with the wild-type (WT). The delivered regulomes (Wang et al. 2018, Wu et al. 2020) differ substantially as they were acquired under different conditions, and foremost, at various growth phases. Further data on the Mhy1’s regulome and coregulome with Rim101 were delivered by Shu et al. (2021). That study specifically focused on alkaline pH-induced filamentation; thus, both the TFs were relevant. In relation to Wu et al. (2020), Mhy1–Rim101-coregulated cell wall proteins were identified and coined as key effectors of the dimorphic transition. The cooperation of Mhy1 and Rim101 during pH-dependent filamentation was evidenced. In contrast, the decoupling of the Rim101-governed pH-response mechanism and Hoy1-driven filamentation was proved by the morphogenetic shift being still operable in the *Δrim101* background (Szabo and Štofaníková 2002). The authors provided evidence that dimorphic transition is primarily dependent on the type of N source, and organic N is required. AA import is dependent on the ambient pH, the common point of convergence of the two TFs. In this sense, the Hoy1-regulated filamentation could be interpreted as pH/Rim101-dependent, but are mechanistically independent. Indeed, *HOY1*’s promoter comprises stress response and N-regulatory cis-elements, indicating that Hoy1 is activated in response to general stress, specifically to AA starvation, but is pH-independent.

In the context of the gTME strategy, the in-depth study of Rim101–Mhy1/Hoy1 exemplifies the complexity of TRNs, their potential overlap, the importance of considering coregulators, and a way to technically address this issue.

### TFs involved in lipid metabolism

The second most studied complex trait in *Y. lipolytica* is the propensity for the assimilation, synthesis, and accumulation of lipids (Beopoulos et al. 2009, Nicaud 2012, Trébulle et al. 2017). The work by Trébulle et al. (2017) can be considered a compendium of the lipid metabolism-related TRN in *Y. lipolytica*. The authors recycled the data available in extensive repositories and reconstructed a gene regulatory network comprising 111 TFs; 4451 target genes; and 17 048 regulatory interactions (Trébulle et al. 2017). Experimental evidence was provided for a few of the TFs included in the reconstructed network.

Involvement in lipid metabolism in *Y. lipolytica* was proved for, *i.a*. Por1/*D12628* (Poopanitpan et al. 2010); Mig1/*E07942* (Wang et al. 2013); Yas1/*C02387* and Yas2/*E23287* (Endoh-Yamagami et al. 2007); Yas3/*F18590* (Hirakawa et al. 2009); and Gzf2/*F17886* and Gzf3/*C22682* (Pomraning et al. 2018). The Yas family mediates responses to alkane provision via the induction or repression of downstream genes. In contrast, Gzfs were specifically involved in N catabolite repression (NCR; responsive to the type and level of the N source available), being only indirectly associated with lipid accumulation. As evidenced, the disruption of N-utilization regulators elevated lipid accumulation, by reducing the expression of the β-oxidation pathway (Pomraning et al. 2017). A direct implication of Por1 in lipid catabolism was proven by studies on a *Δpor1* mutant, defective in the synthesis of various fatty acids, transcriptional induction of genes involved in β-oxidation, and peroxisome proliferation. Similarly, the OE of *DEP1/F05896* enhanced lipid accumulation in *Y. lipolytica* (Leplat et al. 2018). Contradictory results were obtained for the Dep1 homologs from *S. cerevisiae* and *Fusarium* spp. In the former (Lamping et al. 1994), it was identified as a repressor of phospholipid biosynthesis-related genes, unlike in the latter (Zhang et al. 2020). These pioneering studies suggest a higher similarity between the *Y. lipolytica* Dep1 (ylDep1) and that of *Fusarium*. Direct comparisons of *DEP1*-OE/KO in *Y. lipolytica* (Gorczyca et al. 2024) did not assess their influence on lipid metabolism, but on another complex trait, namely rProt biosynthesis (discussed hereafter). The deletion of *MHY1*, with a known role in the dimorphic transition, contributed to enhanced lipid accumulation, via increased C flux through lipid biosynthesis (Wang et al. 2018). This result suggested either a specific mechanism by which dimorphic transition is executed and/or pleiotropic functions of Mhy1 in *Y. lipolytica*.

Based on sequence similarity, ylMig1 was deemed to be involved in glucose-related repression by the negative regulation of transcription. In *S. cerevisiae*, Mig1 was involved in invasive growth (Yang et al. 2018). Functional studies on the *Δmig1* phenotype revealed that Mig1 is involved in lipid metabolism in *Y. lipolytica*, as the mutants had more lipid bodies and higher lipid content (Wang et al. 2013). Omic studies indicated that the enhanced expression of multiple genes involved in lipid biosynthesis, and the downregulation of *MFE1* involved in β-oxidation, explaining the biochemical and molecular mechanisms behind this phenotype (Wang et al. 2013). Direct comparisons of *MIG1*-OE and -KO in *Y. lipolytica* revealed no effect on growth or rProt synthesis (Gorczyca et al. 2024), and its OE did not alter the lipid content (Leplat et al. 2018).

### Specific factor-response TFs

Several ylTFs were identified as responsive to specific external factors. One such TF is Crf1/*B08206*, a Cu^2+^-binding transcriptional regulator, responsive to the concentration of this ion (García et al. 2002a). Functional studies revealed that the *Δcrf1* strain is up to 5-fold more resistant to Cu^2+^. Interestingly, ylCrf1 is not regulated at the transcriptional level by the addition of Cu^2+^; Crf1 is relocalized to the nucleus during growth in a Cu-supplemented medium (García et al. 2002b). The high specificity of Crf1 toward Cu^2+^ was exploited for the construction of a *CRF1*-cassette, enabling dominant selection during the genetic engineering of *Y. lipolytica* (Guo et al. 2011). The OE of *CRF1* in *Y. lipolytica* slightly increased growth and rProt synthesis under a low pH of 3.0 (Gorczyca et al. 2023). The importance of the external pH in copper detoxification is well-known and provides a link between the observations made in *Δcrf1* and *OE-CRF1*. Additionally, the OE of *CRF1* marginally enhanced the strain’s resistance to 0.25 mg ml^−1^ menadione, which is an oxidative stress inducer, and increased the lipid contents in *Y. lipolytica* (Gorczyca et al. 2023). Consistently, a similar lipid-accumulation-promoting effect (85%) was also observed in another *OE-CRF1 Y. lipolytica* strain (Leplat et al. 2018). In summary, although the Crf1-TRN’s activator is very specific, the downstream impacts are pleiotropic, which illustrates the essence of gTME.

Another TF, whose activity is induced by a specific external factor is Rim101 (Lambert et al. 1997). YlRim101 is involved in pH response. Specifically, under an alkaline pH, the C-terminal of Rim101 is proteolytically cleaved, and the truncated form activates the transcription of the genes expressed at an alkaline pH and represses the transcription of those expressed at an acidic pH (compare Fig. 2). The OE of this truncated turns its transcriptional function to be constitutive, irrespective of the ambient pH. Moreover, Shu et al. (2021) demonstrated that Rim101 is specifically involved in the regulation of alkalinity-induced filamentation. The major dimorphic shift regulator, Mhy1, governs both alkalinity- and glucose-induced filamentation; *Δmhy1* abolishes but *MHY1* OE induces filamentation, irrespective of the conditions. Rim101 KO/OE impairs/induces filamentation at alkaline/acidic pH, respectively, proving its primary role in ambient pH response, but only a secondary role in filamentation. A study by Shu et al. (2021) provides a detailed functional description of Rim101, including its regulome, and signaling mechanism. Notably, Rim101-OE markedly suppressed the synthesis and secretion of rProts, especially at a higher pH; 7 vs 5 (Celińska, unpublished).

Slight pH responsiveness was also observed for a zinc finger Msn2/4 family representative in *Y. lipolytica*. Msn4/*C13750* promoted tolerance to acid-induced stress, as deletion mutants displayed growth defects at pH 3, while its OE reduced cell chain formation (Wu et al. 2020). *MSN4*-OE contributed to a minor induction of growth under different culture conditions during high-throughput screens (Gorczyca et al. 2024). Its expression was upregulated due to UPR, showing that the stress factor associated with Msn4-driven stress response may also be of internal origin (Korpys-Woźniak and Celińska 2021). Such an observation is specifically relevant to the gTME-driven promoting effects of *HAC1-MSN4* OE on rProt production in *P. pastoris* (Zahrl et al. 2023). Overall, based on its similarity to *S. cerevisiae*, Msn4 may presumably perform multiple pleiotropic functions in *Y. lipolytica*, which are not yet directly studied.

The primary report on TF Euf1/*F01562* suggested its 10-fold upregulation, specifically in response to erythritol (Rzechonek et al. 2017), promising to be a very potent and precise tool for gTME. The initial discovery of Euf1 indicated it as a regulator of the Erythritol Utilization Gene Cluster (Rzechonek et al. 2017, Mirończuk et al. 2018). Further studies focused on the characterization of its regulome via comparative transcriptomics (Rzechonek et al. 2024). They revealed Euf1’s targets and conditions that must cooccur to elicit the expression, including- (i) Euf1 should be operable, (ii) erythritol has to be present, and (iii) the culture medium must be carbon source deficient. The report by Celińska et al. (2024) confirmed all these mechanisms, shedding extra light on a Euf1-driven transcriptional programme. It was evidenced that Euf1-targeted transcription can be also initiated by osmotic stress induced by sorbitol, a nonconsumable osmoactive compound. Yet, the regulator of *EUF1* expression itself has not been established; which would be of great interest to gTME. By reanalyzing the RNAseq data acquired by Celińska et al. (2024), Rzechonek et al. (2024) discovered that erythritol is indeed not the sole inducer of *EUF1* expression (as was inferred) but the lack of other carbon sources is crucial. The mechanism involved is driven by another TF, Adr1/*F21923*. Based on sequence similarity, Adr1 is considered a carbon-source-responsive inducer of glucose-repressed genes, promoting the utilization of alternative carbon sources. Celińska et al. (2024) proposed a mechanism involving the coordinated action of Euf1–Adr1 on the Euf1-dependent regulome, explaining the corequirement of the three aforementioned conditions. Additionally, an interesting pattern of *EUF1* transcript splicing and dimerization was proposed, which is promoted, but not conditioned, by erythritol (Celińska et al. 2024). Such a molecular circuit may be valuable for devising gTME strategies.

While Hac1/*B12716* is known for its pleiotropic functions, its activation depends on a specific factor—exposure of hydrophobic, misfolded polypeptides in the ER lumen. The exact mechanisms by which Hac1 operates and the effectors that sense and perform activating splicing were described in detail in different yeast spp., including *Y. lipolytica* (Guerfal et al. 2010, Oh et al. 2010). The Hac1-governed TRN mediates the activation of hundreds of molecular events, including the enhanced provision of chaperones and membranes, to concertedly relieve the overloaded secretory pathway, making it specifically interesting for gTME (Guerfal et al. 2010, Oh et al. 2010, Callewaer et al. 2011, Hooks and Griffiths-Jones 2011, Whyteside et al. 2011). Indeed gTME strategies with Hac1 have been implemented in many yeast spp., including *Y. lipolytica* (discussed hereafter for *Y. lipolytica*) (Gasser et al. 2007a, b, Graf et al. 2009, Zahrl et al. 2017, 2019, Korpys-Woźniak et al. 2021, Korpys-Woźniak and Celińska 2023). Studies into the fundamentals of Hac1 operation in *Y. lipolytica* showed that the activation of its expression strongly depends on the biochemical characteristics of the overproduced rProt and its folding (Korpys-Woźniak and Celińska 2021). Investigation into the Hac1’s regulome (Korpys-Woźniak and Celińska 2023) indicated that elevated *HAC1* expression modulates ribosome biogenesis, nuclear and mitochondrial events, cell cycle, gene expression catalyzed by RNA polymerases III and II, proteolysis, and RNA metabolism in *Y. lipolytica*. Specific downstream targets of Hac1 in *Y. lipolytica* were identified, further clarifying its mechanism of action. In addition, the posttranscriptional regulation of *HAC1* (unconventional and activating splicing) was investigated. In strains suffering from UPR, the *HAC1* splicing rate was only slightly higher in *Y. lipolytica*, ascertained based on their global transcriptome. Practical outcomes of Hac1’s TRN use for the rProt synthesis engineering are addressed hereafter; e.g. gTME in *Y. lipolytica*.

A six-member family of Gzf TFs is specifically deregulated by the type of N source. Pomraning et al. (2017) unraveled the complex TRN of the Gzfs and their mutual interactions. The regulomes for Gzf2/3 were also described. Transcription of *GZF1*/*D20482, GZF2*/*F17886, GZF4*/*E05555*, and *GZF5*/*E16577* was activated when *Y. lipolytica* was grown on ammonium, with the highest amplitude observed for *GZF1*; hence termed “inducers of NCR.” However, the *Δgzf1* genotype displayed no growth defect on any N source (Pomraning et al. 2017). While *GZF2*/*4* were upregulated in *Y. lipolytica* strains overproducing rProts, *GZF1* was identified among the most strongly downregulated genes, which was counter-intuitive, considering its N scavenging activity (Korpys-Woźniak and Celińska 2021). A phenotypic investigation of the *Δgzf3* (*C22682*) strain by Pomraning et al (2017) revealed that Gzf3 regulates the N-dependent activity of Gzf1. While the mechanism of action of Gzf1 is the most complex and the least understood, this specific TF turned out to be of particular interest for gTME due to the confirmed, universal enhancement of rProt synthesis upon its OE (Gorczyca et al. 2023, 2024).

### Guilt-by-association-and/or-similarity TFs

A wide set of TF-encoding genes, identified in the *Y. lipolytica* genome via a specific domain search, were ascribed a specific name and putative function due to their expression profile and/or sequence similarity to other yeast counterparts, without functional experimental evidence. While such an approach is a common practice, greatly fostering knowledge gain, its application must be always considered carefully.

Such a group of TFs is represented by global regulators involved in carbon/nitrogen metabolism, i.a. *ERT1/*/*E18304/E03410, DAL81*/*D02783, GCN4*/*E27742, ADR1*/*F21923, CAT8*/*C19151*, and *AZF1*/*A16841*. Based on sequence similarity search and cross-referencing several databases (Gorczyca et al. 2024) *ERT1* was identified as a positive regulator of fermentable carbon utilization, technically repressing RNA polymerase II by a nonfermentable carbon source. Expression of *ERT1-1/*/*E18304* was induced under the high-level rProt synthesis, and OE-enhanced rProt synthesis (Gorczyca et al. 2024) and reduced lipid accumulation (Leplat et al. 2018). A protein sequence similarity search identified *D02783* as *DAL81*, positive regulators of multiple N degradation pathways. Its expression level was not changed due to rProt OE (Korpys-Woźniak and Celińska 2021) but exerted a positive influence on rProt synthesis upon OE. In contrast, another N-metabolism-related gene, *GCN4*, triggered a minuscule decrease in rProt synthesis levels in *Y. lipolytica* upon OE (Gorczyca et al. 2024). While counter-intuitive, as Gcn4 responds to AA starvation and induces the expression of AA biosynthesis-related genes, consistent with the observations in *S. cerevisiae* (Mittal et al. 2017). These authors explained that *GCN4*-OE diminished protein synthesis capacity via the negative regulation of ribosomal proteins.

Similar to the aforementioned Adr1 (identified by a homology search), the TF Cat8/*C19151* is implicated in the assimilation of nonglucose carbon sources. It regulates gluconeogenesis and the glyoxylate cycle. However, per the current model, Cat8 activates while Adr1 derepresses the downstream genes under glucose deprivation. Though, its expression was not deregulated due to rProt synthesis (Korpys-Woźniak and Celińska 2021), its OE triggered a decline in rProt production, particularly markedly under high OA (Gorczyca et al. 2024). The biological sense seems to be understandable, upon starvation, rProt synthesis is indirectly turned off as it is an energy- and resource-consumptive process (Kubiak-Szymendera et al. 2022). Cat8 was irrelevant to lipid metabolism (Trébulle et al. 2017, Leplat et al. 2018); however, Kerssemakers et al. 2023a, b) proved its transcriptional deregulation in response to O_2_ availability, and by delimiting its iModulon, its involvement in respiration and energy homeostasis regulation under reduced O_2_ availability.

The TF *D07744* is weakly similar to an AP-1-like TF from *Neurospora crassa*. It is a basic leucine zipper (bZIP) domain-containing TF, having a structure typical for a yeast activator protein (YAP). In *S. cerevisiae*, the YAP subfamily is composed of eight members (YAP1–8), which are involved in environmental stress response (Rodrigues-Pousada et al. 2019): Yap1 is the major regulator of oxidative stress response; is involved in Fe metabolism (like Yap5) and the detoxification of arsenate (like Yap8). Yap2 is associated with cadmium stress responses, while Yap4 and Yap6 play a role in osmotic stress response. The *Y. lipolytica* ortholog YAP-like/*D07744* demonstrated a significantly enhanced expression during a dimorphic shift induced by alkalinization of the ambient pH (Morales-Vargas et al. 2012). Consistently, elevated pH promoted the growth of a *D07744*-OE *Y. lipolytica* strain (Gorczyca et al. 2020). This TF’s OE also enhanced oxidative stress resistance and lipid accumulation (Gorczyca et al. 2023). In contrast, Leplat et al. (2018) did not observe any alterations in lipid accumulation upon *D07744*-OE.

Azf1 is a carbohydrate-sensing TF, which in the presence of glucose, activates genes involved in growth, carbon metabolism, and cell wall organization in *S. cerevisiae* and *O. polymorpha* (Stein et al. 1998, Semkiv et al. 2022). Consistently, the OE of a similar Azf1/*A16841* in *Y. lipolytica* enhanced cell-wall remodeling, and the “carbon type” used in the growth medium contributed markedly to the phenotype of this strain (Gorczyca et al. 2024). *AZF1*-OE triggered a dramatic decrease in rProt synthesis, however, a *Δazf1* mutation rendered no aberrant phenotype in this regard. Moreover, when grown on glucose, the OE of *AZF1* contributed to the significant decrease in lipid content (26.6%) in *Y. lipolytica* (Leplat et al. 2018). Such an effect was not observed when glycerol was employed as the main carbon source, indicating a specific role of glucose in the Azf1 TRN, and providing experimental evidence for the correct identification of *A16841* as an Azf1.

Though the implication of *SKN7*/*D14520* in osmotic stress response in *Y. lipolytica* is widely acknowledged, no direct functional studies have been conducted until recently. YlSkn7 sequence shows weak similarity (<30%) to the Skn7 from *S. cerevisiae*, known to be associated with protein secretion and the activation of oxidative and osmotic stress responses (Rep et al. 2001, Karlgren et al. 2004). Its specific role in *Y. lipolytica* was elucidated in a series of recent reports. The first indication was that *D14520* was remarkably upregulated in *Y. lipolytica* in response to hyperosmolality (Kubiak-Szymendera et al. 2022). Then, the exposure of *Δskn7 Y. lipolytica* to hyperosmotic conditions abolished growth, while its OE enabled maintaining r-Prot synthesis capacity under stress conditions (Gorczyca et al. 2023). Interestingly, the genotype *Δhsf1* displayed severely impaired growth under both “stressful,” and “optimal” growth conditions; suggesting that the TRN of Skn7 is narrower. The OE or KO of *SKN7* did not render any influence on lipid levels in *Y. lipolytica* (Leplat et al. 2018, Gorczyca et al. 2023).

## gTME in *Y. lipolytica*

A conventional, rationally designed gTME strategy starts with the identification of a TF and its implication in a biological process of interest. While extensive knowledge about a specific TRN is not required, it improves predictability and facilitates the design of a detailed cloning strategy. Identification may be executed by a YALI number search (using the information on *Y. lipolytica*), AA sequence similarity search (using data on other yeast spp.), or genome scanning for the occurrence of specific motifs. YALI number search is now greatly facilitated by the wide availability of data in repositories and search tools. Specifically in terms of TF search in *Y. lipolytica*, a dedicated database YaliFunTome (https://sparrow.up.poznan.pl/tsdatabase/) has been created (Gorczyca et al. 2024). It covers all the TFs identified in *Y. lipolytica*’s genome utilizing genome sequence scanning for TF-specific motifs encoding typical domains, like zinc fingers, C2H2, Zn(2)-Cys(6), bZIP, and HLH (Leplat et al. 2018).

The original gTME comprises application-oriented studies, where TFs were used as tools for global adjustment of a strain’s performance concerning specific characteristics, usually complex traits. As summarized in Section 2, such approaches have been executed in other yeast species with much success. In *Y. lipolytica*, several examples of gTME have been reported. The following sections present these cases and summarize the outcomes.

### Global increase in lipid accumulation

The pioneering studies using gTME engineering in *Y. lipolytica* involved creating a library of strains, individually OE a single TF (Leplat et al. 2018), to experimentally identify the TFs involved in lipid accumulation. To technically execute this ambitious idea, a high-throughput transformation method was first developed (Leplat et al. 2015). The resultant strains were subjected to high-throughput OE screens in glucose and glycerol-based media yielding the first gTME project data in *Y. lipolytica* (Leplat et al. 2018).

Overall, these screens identified 38 TFs impacting lipid accumulation in *Y. lipolytica*, with three of them (Hoy1, Gzf3, and TFIIIA/*F05104*) being identified as vital, consistent with what was deduced from the computational method (Trébulle et al. 2017). The carbon source type was a significant variable impacting the TF-driven lipid-accumulation-promoting phenotype. In the glucose medium, among the 26 strains with differential phenotypes, the most prominent changes ranged from Azf1/*A16841* (–26.6%) to Brg1/*E31757* (+90.9%). In glycerol medium, the total number of strains with altered lipid contents was 20, from −55.7% for Hoy1/*A18469* to +74% for Rpn4/*F25861*. The universal enhancers: *C13178*, Rei3/*B08734*, and Gzf3/*C22682*, and repressors: Hoy1/*A18469* and Msn4/*C13750* of lipid synthesis were identified. The TF Por1/*D12628*, previously identified as an activator (Poopanitpan et al. 2010), was also found among the TFs promoting lipid accumulation in glycerol medium (Leplat et al. 2018). Likewise, Dep1/*F05896* implication in this trait (Zhang et al. 2020) was confirmed by elevated lipids accumulation by >20% in those screens (Leplat et al. 2018). These TFs may be considered the first choice candidates for gTME targets for enhanced lipid biosynthesis in *Y. lipolytica*.

### Global enhancement of protein synthesis

Another trait engineered in *Y. lipolytica* via gTME was the synthesis of proteins, with a particular focus on rProts. *Yarrowia lipolytica* has emerged as an efficient rProt production platform, and the molecular background of this propensity has been reviewed (Celińska and Nicaud 2019). Presented in a recent series of papers, efforts toward elevating the yield of rProt via gTME prove the feasibility of such an approach. All the data can be easily accessed and browsed, as they have been deposited in the YaliFunTome database.

Apart from TFs that promote rProt synthesis in a condition-specific manner, several TFs were identified as “global enhancers”—potent to develop the desired phenotype, irrespective of the conditions. To our understanding, these are specific targets for gTME. Among these was TF Klf1/*D05041*, a zinc finger TF Krueppel-like factor (a name assigned based on sequence similarity) (Gorczyca et al. 2023, 2024). Klf1 was extensively studied in *Schizosaccharomyces pombe* during long-term G0 quiescence induced by N starvation (Shimanuki et al. 2013). Expression of *KLF1* was induced under N starvation, while ∆*klf1* mutants exhibited changes in cell morphology and were unable to restore cell division. It specifically reacts to oxidative stress via nuclear relocalization (Herholz et al. 2019). The regulome of the schpKlf1 covers genes implicated in cell wall renewal, oxidative stress response, glycolysis, nutrient uptake, RNA-mediated chromatin silencing, glycosidation, and methylation (Shimanuki et al. 2013). When applied as a gTME tool in *Y. lipolytica*, the rProt synthesis capacity was greatly promoted, frequently by >2-fold. Such an effect was significantly impacted by external factors like high O_2_, organic N source, enhanced temperature, and lower pH (Gorczyca et al. 2023, 2024); the promoting effect of *KLF1*-OE was observed despite external conditions. Interestingly, *KLF1*’s expression did not change in response to OE of different rProts (Korpys-Woźniak and Celińska 2021, Gorczyca et al. 2024), suggesting its activation via posttranslational modification.

Likewise, the OE of *GZF1*/*D20482* triggered a universal increase (∼1.2–2-fold) in rProt synthesis (Gorczyca et al. 2023, 2024). The same set and levels of external factors as with Klf1, enhanced the beneficial effect of *GZF1*-OE on rProt synthesis in *Y. lipolytica*. Yet, consistently with its role in NCR, the provision of organic N source had lesser importance for the production of rProt (versus control). Intriguingly, the rProt synthesis-promoting influence was not observed with the other NCR inducer from the Gzf family, Gzf4/*E05555* (Pomraning et al. 2017). In concordance, the transcriptional profile of *Y. lipolytica* strains producing different rProts (Korpys-Woźniak and Celińska 2021) and their effects on rProt synthesis (Gorczyca and Celińska 2024, Gorczyca et al. 2024) indicate that Gzf1 and Gzf4 are not redundant, and each plays a specific and distinct role. Considering Gzf1’s role as an NCR activator, its rProt synthesis-promoting impact can be attributed to enhanced N scavenging capacity.

Expectedly, *HSF1/E13948*, a key regulator of global stress response and a major activator of the cell’s folding and chaperoning capacity (Lindquist and Craig 1988) was identified to be another “global enhancer” of rProt synthesis (Gorczyca et al. 2024), though it did not display any remarkable transcriptional deregulation profile due to rProt production (Korpys-Woźniak and Celińska 2021, Gorczyca et al. 2024). As discussed in Section 2, Hsf1 was one of the most frequently utilized tools in gTME for enhanced rProt production in yeast. In contrast to the observations in *KLF1*-OE and *GZF1*-OE *Y. lipolytica, HSF1*-OE promoted rProt production even at the expense of growth; rProt was inversely associated with growth (growth–rProt correlation; *r* < 0.45), acting very specifically on this particular complex trait. A similar phenomenon—growth arrest upon synthetically pushed rProt synthesis—was observed and quantitatively described in terms of substrate utilization and C channeling (Gorczyca et al. 2022).

Based on similarity to Hsf1, the use of Hac1/*B12716* as a gTME tool for managing UPR was expected to massively improve rProt synthesis and secretion. Yet, the effect of *HAC1*-OE in *Y. lipolytica* was predominantly negative for an intracellular rProt (Gorczyca et al. 2024). However, the result varied when a secretory rProt was used as a reporter. The overall gain in secretory rProt synthesis was mediocre, ∼30% (Korpys-Woźniak et al. 2021). However, more in-depth studies demonstrated that the impact was indeed bifurcated, causing a nearly 7-fold drop in the retained fraction of rProt but promoting its secretion by nearly 2.5-fold (Korpys-Woźniak and Celińska 2023), which makes ylHac1 a potent gTME tool when rProt secretion is a specific bottleneck.

### Engineering resistance to technical limitations of bioprocesses

#### Growth under oxygen limitation

It is well known that insufficient O_2_ provision is the primary technical limitation in industrial bioprocesses based on *Y. lipolytica*; its reaction to limited O_2_ supply was studied in detail (Kamzolova et al. 2003, Bellou et al. 2014, Jost et al. 2015, Ferreira et al. 2016, Timoumi et al. 2017, Kerssemakers et al. 2023a, b). Different bioprocess technologies and genetic engineering strategies were employed to overcome this limitation (Bhave and Chattoo 2003, Lopes et al. 2009, Mirończuk et al. 2019); yet, further improvement is needed. TFs enabling the sustained growth of *Y. lipolytica* under fluctuations and gradients in O_2_ provision would be of great interest.

The greatest expectations in this regard were related to the use of Hap1/*F17424*, an ortholog of *S. cerevisiae*’s heme activating protein, known to promote respiratory metabolism and growth (Zhang and Hach 1999). *HAP1*-OE triggered a marked increase in the rProt synthesis capacity of *S. cerevisiae* (Martínez et al. 2016). In contrast, *HAP1*-OE in *Y. lipolytica* made it remarkably more sensitive to limited O_2_ supply, as the strain suffered from significant growth limitation under low aeration (Gorczyca et al. 2024). Nonetheless, other TFs promoting growth (≥80%) during O_2_ limitation, including Lac9/*D20460*, Jmc2/*B14443, B20944*, and Dal81/*D02783* were identified. Their specific function in *Y. lipolytica* has not been studied, and the names were assigned based on sequence similarity. Nevertheless, considering the significant practical implications of identifying “anoxia-resistance factors” in *Y. lipolytica*, further functional studies are imperative.

#### Independence from a high-cost nitrogen source

The cost of organic N sources constituted the highest unit production expenditure in a pilot-scale rProt production process model (Kubiak et al. 2021). Therefore, it is of high interest to identify a factor that would enable efficient rProt synthesis in a medium composed of cheaper N sources, like inorganic ammonium salts.

As stated above, due to the architecture of its promoter, the expression of *Hoy1*/*A18469* is activated in response to N starvation. While identified as dimorphic-transition-related TF, Hoy1 appears to be specifically sensitive to N provision. Hoy1’s abundance forced via OE, triggered a remarkable elevation in rProt production under organic N limitation (Gorczyca et al. 2024). As the ylHoy1 regulome is not yet known, any mechanistic explanation of its action is only speculative. It seemed that Hoy1-OE facilitated N capture under scarcity, contributing to enhanced rProt production. Indeed, N was one of the variables with a significant impact in shaping the *HOY1-*OE strain’s phenotype. It would be interesting to ascertain the universality of this phenomenon by studying different reporter proteins and media compositions.

## Summary and perspectives

While less explored when compared to *S. cerevisiae*, gTME in *Y. lipolytica* seems to be a potent approach to engineering complex traits. The exemplary outcomes of gTME execution in *Y. lipolytica* highlight the complex traits/functionalities that have been targeted, so far. Knowledge of ylTFs, summarized in Section 3, provides indices for matching a TF or a TRN to a desired functionality. Considering the current trends in yeast research, and the technical feasibility of high-throughput cloning, a reliable, well-validated high-throughput cultivation/analysis technique, tailored for *Y. lipolytica* is needed to take full advantage of the gTME approach. It is specifically crucial when engineering TRN, due to the pleiotropic functions of TFs. A TRN-engineered strain may most probably develop relevant phenotypes under varying environmental perturbations. Such a protocol, and a revision of the other with an evaluation of their pros and cons, has been recently proposed (Celińska and Gorczyca 2024).

## Footnotes

1 A gene name is indicated in capital letters and italicized. A protein name is indicated without capitalization and italicization.

2 Throughout the text, the gene signatures refer to YALI0 numbers as assigned originally to the first version of *Y. lipolytica* genome (Dujon et al. 2004); the prefix “YALI0” is skipped.
